# Marmosets as Research Models of Alzheimer's Disease (MARMO‐AD)

**DOI:** 10.1002/alz70856_100186

**Published:** 2025-12-24

**Authors:** Afonso C Silva, Jung Eun Park, Gregg E Homanics, Lauren K Hayrynen Schaeffer, Julia Oluoch, Nicholas T Seyfried, David J Schaeffer, Amantha Thathiah, Gregory W Carter, Stacey J Sukoff Rizzo

**Affiliations:** ^1^ University of Pittsburgh School of Medicine, Pittsburgh, PA, USA; ^2^ Emory University School of Medicine, Atlanta, GA, USA; ^3^ The Jackson Laboratory for Genomic Medicine, Farmington, CT, USA; ^4^ The Jackson Laboratory, Bar Harbor, ME, USA

## Abstract

**Background:**

In 2022, with funding from the National Institute on Aging, we established a new consortium to generate, characterize, and validate MArmosets as Research MOdels of Alzheimer's Disease (MARMO‐AD). This consortium develops and studies gene‐edited marmoset models that carry genetic mutations causative of AD, comparing them to wild‐type aging marmosets from birth throughout their lifespan. Creating a nonhuman primate model of AD is a crucial step toward overcoming the limitations of other model systems and essential for investigating primate‐specific mechanisms underlying the cellular and molecular root causes of the pathogenesis and progression of Alzheimer's disease.

**Method:**

MARMO‐AD is a lifespan study of our gene‐edited marmoset AD models that employs non‐invasive longitudinal assessments. We utilized CRISPR/Cas9 to create marmosets with C410Y or A426P point mutations in PSEN1, which are causative for familial AD. Founders and their germline offspring undergo comprehensive longitudinal studies using non‐invasive measures, including behavior, biomarkers, neuroimaging, and multiomics signatures. We characterize the genetic, molecular, functional, behavioral, cognitive, and pathological features of aging and AD.

**Result:**

The consortium successfully generated viable founders with C410Y‐ and A426P‐PSEN1 mutations. Germline transmission was demonstrated in the line from C410Y. Before adulthood, PSEN1 mutation carriers showed increases in plasma amyloid beta compared to non‐carriers. Brain analysis revealed alterations in several enzyme‐substrate interactions within the gamma‐secretase complex. The longitudinal characterization of these models, their germline offspring, and normally aging outbred marmosets is ongoing. All data and resources from this consortium will be shared with the broader Alzheimer's disease research community.

**Conclusion:**

By developing marmoset models of Alzheimer's disease (AD), we can explore the distinct cellular and molecular factors unique to primates that contribute to the onset and progression of AD. This approach will help us address the limitations associated with other model organisms and facilitate future translational studies aimed at accelerating the development of therapies for patients.

